# l-Rhamnose Metabolism in Clostridium beijerinckii Strain DSM 6423

**DOI:** 10.1128/AEM.02656-18

**Published:** 2019-02-20

**Authors:** Mamou Diallo, Andre D. Simons, Hetty van der Wal, Florent Collas, Bwee Houweling-Tan, Servé W. M. Kengen, Ana M. López-Contreras

**Affiliations:** a Wageningen Food and Biobased Research, Wageningen, The Netherlands; b Laboratory of Microbiology, Wageningen University and Research, Wageningen, The Netherlands; University of Tartu

**Keywords:** 1,2-propanediol, IBE fermentation, l-rhamnose, *Ulva lactuca*, propionic acid

## Abstract

Macroalgae (or seaweeds) are considered potential biomass feedstocks for the production of renewable fuels and chemicals. Their sugar composition is different from that of lignocellulosic biomasses, and in green species, including Ulva lactuca, the major sugars are l-rhamnose and d-glucose. C. beijerinckii DSM 6423 utilized these sugars in a U. lactuca hydrolysate to produce acetic acid, butyric acid, isopropanol, butanol, and ethanol (IBE), and 1,2-propanediol. d-Glucose was almost completely consumed in diluted hydrolysates, while l-rhamnose or d-xylose was only partially utilized. In this study, the metabolism of l-rhamnose by C. beijerinckii DSM 6423 was investigated to improve its utilization from natural resources. Fermentations on d-glucose, l-rhamnose, and a mixture of d-glucose and l-rhamnose were performed. On l-rhamnose, the cultures showed low growth and sugar consumption and produced 1,2-propanediol, propionic acid, and *n*-propanol in addition to acetic and butyric acids, whereas on d-glucose, IBE was the major product. On a d-glucose–l-rhamnose mixture, both sugars were converted simultaneously and l-rhamnose consumption was higher, leading to high levels of 1,2-propanediol (78.4 mM), in addition to 59.4 mM butanol and 31.9 mM isopropanol. Genome and transcriptomics analysis of d-glucose- and l-rhamnose-grown cells revealed the presence and transcription of genes involved in l-rhamnose utilization and in bacterial microcompartment (BMC) formation. These data provide useful insights into the metabolic pathways involved in l-rhamnose utilization and the effects on the general metabolism (glycolysis, early sporulation, and stress response) induced by growth on l-rhamnose.

**IMPORTANCE** A prerequisite for a successful biobased economy is the efficient conversion of biomass resources into useful products, such as biofuels and bulk and specialty chemicals. In contrast to other industrial microorganisms, natural solvent-producing clostridia utilize a wide range of sugars, including C_5_, C_6_, and deoxy-sugars, for production of long-chain alcohols (butanol and 2,3-butanediol), isopropanol, acetone, *n*-propanol, and organic acids. Butanol production by clostridia from first-generation sugars is already a commercial process, but for the expansion and diversification of the acetone, butanol, and ethanol (ABE)/IBE process to other substrates, more knowledge is needed on the regulation and physiology of fermentation of sugar mixtures. Green macroalgae, produced in aquaculture systems, harvested from the sea or from tides, can be processed into hydrolysates containing mixtures of d-glucose and l-rhamnose, which can be fermented. The knowledge generated in this study will contribute to the development of more efficient processes for macroalga fermentation and of mixed-sugar fermentation in general.

## Abstract (precis)

A prerequisite for a successful biobased economy is the efficient conversion of biomass resources into useful products, such as biofuels and bulk and specialty chemicals. In contrast to other industrial microorganisms, natural solvent-producing clostridia utilize a wide range of sugars, including C_5_, C_6_, and deoxy-sugars, for production of long-chain alcohols (butanol and 2,3-butanediol), isopropanol, acetone, *n*-propanol, and organic acids. Butanol production by clostridia from first-generation sugars is already a commercial process, but for the expansion and diversification of the acetone, butanol, and ethanol (ABE)/IBE process to other substrates, more knowledge is needed on the regulation and physiology of fermentation of sugar mixtures. Green macroalgae, produced in aquaculture systems, harvested from the sea or from tides, can be processed into hydrolysates containing mixtures of d-glucose and l-rhamnose, which can be fermented. The knowledge generated in this study will contribute to the development of more efficient processes for macroalga fermentation and of mixed-sugar fermentation in general.

## INTRODUCTION

The increasing worldwide demand for fuels and chemicals contradicts the diminishing availability of fossil resources, which are currently the main source of these compounds. In past decades, the concept of biorefinery has been established as an alternative to petroleum-based refineries, in which multiple products (energy, fuels, and [high-value] chemicals) are produced from one biomass source (1, 2). Nowadays, the most established biorefineries are based on lignocellulosic biomasses. However, a diversification of biomass resources is needed to ensure sufficient availability and flexibility of processes. Macroalgae have gained attention in recent years as feedstock for production of fuels and chemicals due to the advantages that they show with respect to traditional terrestrial feedstocks for biorefinery: (i) higher productivity (biomass produced per unit of surface) than terrestrial crops, (ii) no competition for arable land, (iii) lower freshwater consumption during cultivation, and (iv) no requirement for fertilizers (3). In addition, macroalgae show a distinctive chemical composition compared to lignocelluloses and terrestrial crops, as some species are rich in carbohydrates, proteins, fatty acids, and/or bioactive components that make them very suitable for biorefinery as sources of multiple valuable products (4, 5). Current developments in new technologies for large-scale seaweed cultivation are expected to result in increased production volumes of biomass at competitive production costs (6).

In the green seaweed Ulva lactuca, d-glucose and l-rhamnose are the main carbohydrates present in the ulvan polysaccharide, and it has been reported that these sugars could be extracted using mild pretreatment conditions (7, 8). In fermentations performed with Clostridium acetobutylicum and Clostridium beijerinckii, using U. lactuca hydrolysates as the substrate, the solvents acetone, butanol, and ethanol (ABE) were produced. Interestingly, C. beijerinckii was also able to produce 1,2-propanediol when grown on l-rhamnose, but not on d-glucose (7). In contrast, C. acetobutylicum did not show any production of 1,2-propanediol and was unable to grow solely on l-rhamnose. C. beijerinckii DSM 6423 (also NRRL B-593, formerly known as Clostridium butylicum NRRL B-593) was able to grow on l-rhamnose as a sole carbon source, producing 1,2-propanediol but also propanol and propionate, in addition to acetic and butyric acids (9). l-Rhamnose utilization is well studied for some microorganisms, such as Escherichia coli and Salmonella enterica serovar Typhimurium (10, 11), and has been described for Clostridium phytofermentans (12). In the last species, l-rhamnose was shown to be converted along a phosphorylated pathway, involving rhamnulose, rhamnulose phosphate (rhamnulose-P), and lactaldehyde as key intermediates. Lactaldehyde is the precursor of the main end product, 1,2-propanediol (12, 13). 1,2-Propanediol is an interesting chemical. Its production has been studied in different microorganisms, including fungi, bacteria, and yeasts (14, 15). The involvement of bacterial microcompartments (BMC) in the catabolism of 1,2-propanediol into *n*-propanol and propionic acid has been described for C. phytofermentans and other organisms as an interesting feature (12, 15). l-Rhamnose metabolism by solventogenic clostridia, however, is not well characterized. Production of 1,2-propanediol, propionate, and propanol by C. beijerinckii suggests that in this solventogenic species, l-rhamnose is converted by a metabolic route similar to that reported for C. phytofermentans.

The aim of this study was to investigate the l-rhamnose metabolism in the solventogenic strain C. beijerinckii DSM 6423. Growth and product formation on d-glucose or l-rhamnose were compared. The pathway for l-rhamnose conversion was reconstructed through analysis of the recently sequenced genome of this strain (16). The gene transcription profiles in cultures grown on d-glucose and on l-rhamnose as sole carbon sources were determined using RNA sequencing, and the differences observed were analyzed with respect to sugar metabolism, early sporulation, and stress response. The results obtained contribute to enhancement of our knowledge about the unique capability of solventogenic clostridia to ferment a variety of carbohydrates into a wide spectrum of products of commercial interest.

## RESULTS

### Fermentation of Ulva lactuca hydrolysate by C. beijerinckii.

The potential of C. beijerinckii for utilization of U. lactuca hydrolysate, containing d-glucose and l-rhamnose as major sugars, was investigated using a hydrolysate prepared according to the method of Bikker et al. (17). Cultures on hydrolysate and on control media containing d-glucose, l-rhamnose, or a mixture of sugars as main carbon and energy sources were grown in serum flasks. The hydrolysate was rich in d-glucose (115 mM) and l-rhamnose (86 mM) and, in addition, contained 28 mM d-xylose. Very low growth was observed in cultures of the pure hydrolysate or hydrolysate supplemented with nutrients as in CM2 medium. Only a small amount of the d-glucose in the hydrolysate was consumed after 144 h of incubation at 37°C, leaving the d-xylose and the l-rhamnose unused in the medium. When the hydrolysates, both pure and supplemented with nutrients, were diluted 1:1 with demineralized water, their fermentability improved. In the cultures on the diluted hydrolysates (DH), the d-glucose was consumed almost completely, whereas approximately 50% of the d-xylose and approximately 20% of the l-rhamnose were consumed. The major products of the last fermentations were butyrate, acetate, isopropanol, and butanol. Low levels of 1,2-propanediol were detected in the cultures on the DH cultures (see Table S1 in the supplemental material).

The major fermentation products in the l-rhamnose control cultures were butyrate and 1,2-propanediol, whereas an IBE fermentation took place on glucose cultures (Table S1). On all the cultures tested, l-rhamnose was only partially utilized. In the cultures with mixtures of d-glucose and l-rhamnose, d-glucose was completely utilized, and the consumption of l-rhamnose was higher than that observed on l-rhamnose-only cultures (Table S1).

The yields of IB(E) produced per d-glucose or d-glucose–d-xylose consumed in the different cultures are shown in Table S1. In this table it can be observed that in the nondiluted hydrolysate cultures the yields obtained are higher than the yields of IBE produced from d-glucose in the control cultures (0.94 and 0.80 in H-*Ulva* and H-*Ulva*+N, respectively, versus 0.72 in d-glucose control cultures). This is also the case for DH-Ulva+N cultures, where the yield of IB produced was 0.82 mM IB per mM of d-glucose and d-xylose consumed. This indicates that in the hydrolysates most probably other carbon sources, such as oligo- or disaccharides, were present that could be utilized to produce solvents by C. beijerinckii.

### Fermentation of l-rhamnose and l-rhamnose–d-glucose mixture.

**FIG 1:**
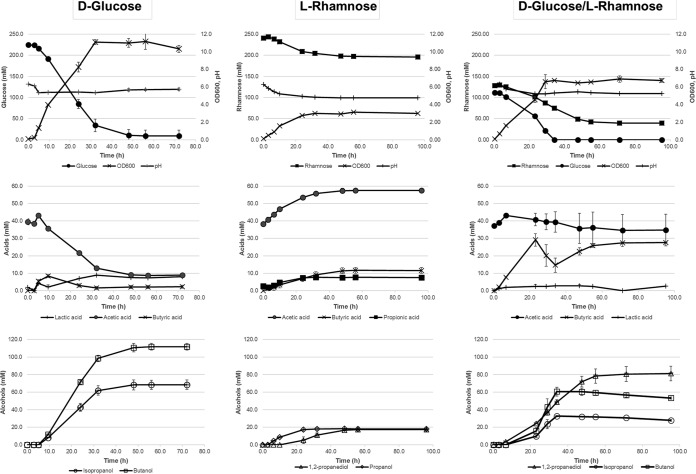
Fermentation profiles of C. beijerinckii grown on d-glucose (left), l-rhamnose (center), and d-glucose/l-rhamnose mixture (right). Fermentations were performed in duplicate in CM2 medium supplemented with the indicated sugars. Only products present at a concentration of >1 mM are shown. SD is shown with error bars, indicating 1 SD of the mean (*n* = 2).

To better characterize the fermentation of l-rhamnose, and to obtain cell material for transcriptome sequencing (RNA-seq) analysis, cultures were grown in bioreactors with 400 ml of working volume without pH control. Samples were taken at different time points for determination of metabolites and for RNA-seq analysis. Fermentations on d-glucose and d-glucose–l-rhamnose mixtures were performed as a reference. The results are shown in Fig. 1 and Table 1. C. beijerinckii was able to grow on l-rhamnose as a carbon and energy source, although growth and l-rhamnose consumption were lower than in d-glucose-grown cultures, with values for optical density at 600 nm (OD_600_) of approximately 3 and 11 for l-rhamnose and d-glucose, respectively (Fig. 1). Growth on l-rhamnose ceased as soon as the pH of the culture dropped below 5, and in contrast to the case of d-glucose-grown cultures, the pH did not increase any more. To check if the growth stopped due to the low pH, a second fermentation on l-rhamnose only was performed, in which the pH was controlled to, or above, 5.2. The growth profile, l-rhamnose consumption, and product formation were not significantly different from those of the non-pH-controlled cultures (results not shown), indicating that the low pH was not the only cause of growth cessation. Interestingly, l-rhamnose fermentation does not lead to the generation of typical solvents like acetone, butanol, and ethanol. Also, reassimilation of acids apparently does not occur, as 19.2 mM acetate and 11.7 mM butyrate were produced in the l-rhamnose culture (Fig. 1; Table 1).

**TABLE 1:** Fermentation data of cultures of C. beijerinckii grown on d-glucose, l-rhamnose, and on a d-glucose–l-rhamnose mixture

Substrate or product	d-Glucose (56 h)	l-Rhamnose (56 h)	d-Glucose/l-rhamnose (72 h)
Substrates at 0 h (mM)			
d-Glucose	224.8		111.2
l-Rhamnose		243.4	129.3
Acetate	39.5	38.3	37.3
Substrates consumed at end (mM)			
d-Glucose	215.3	ND^*a*^	111.2
l-Rhamnose		46.4	86.7
Acetate^*b*^	30.7	0	1.2
Products at end (mM)			
Acetate^*b*^	8.8	57.5 (19.2^*b*^)	36.2
Lactate	7.5	ND	2.4
Butyrate	2.1	11.7	25.9
Acetone	3.2	ND	7.9
Isopropanol	68.5	ND	31.9
Ethanol	6	ND	1.1
Butanol	111.8	ND	59.4
1,2-Propanediol	ND	17.2	78.4
Propanol	ND	18.2	ND
Propionate	ND	7.8	ND
Biomass	26.6	2.1	11.7
Yields and recovery			
Biomass (mol/mol of sugar)	0.12	0.045	0.059
Yield of butanol (mol/mol of d-glucose)	0.52		0.53
Yield of 1,2-propanediol (mol/mol of l-rhamnose)		0.37	0.904
Yield of propanol (mol/mol of l-rhamnose)		0.39	
Yield of propionate (mol/mol of l-rhamnose)		0.17	
Carbon recovery^*c*^ (%)	95	96	88
Electron recovery (%)	98	96	88

a ND, not detected.

b Acetate was produced in l-rhamnose cultures.

c The release of CO_2_ was estimated and included in the calculations.

Instead of producing IBE, as was seen in d-glucose-grown cultures, the strain produced 1,2-propanediol, propanol, and propionate in addition to acetate and butyrate when l-rhamnose was provided as the carbon source. Propionate and propanol are known to be typical products of the catabolism of 1,2-propanediol in many microorganisms, including clostridial species (12).

For biomass determination from the l-rhamnose and on the l-rhamnose–d-glucose cultures, dry matter content was measured at the end of the fermentations. The calibration curve that relates biomass to OD_600_ values of the cultures obtained in d-glucose cultures was not applicable for l-rhamnose-grown cultures, as these show a very different cell morphology (Fig. S1). The highest yields were found for cultures grown on d-glucose or the mixture, with yields of 0.12 and 0.059 mol biomass/mol of sugar consumed, respectively.

As l-rhamnose and d-glucose are both present in hydrolysates from green seaweeds, their cometabolism was studied in cultures grown on a mixture of these sugars in a ratio of 1:1. In Fig. 1 and Table 1, it can be seen that in these cultures all d-glucose was consumed and that the consumption of l-rhamnose was significantly higher than in the rhamnose-only cultures (86.7 mM and 46.4 mM on d-glucose–l-rhamnose cultures and on l-rhamnose, respectively). Interestingly, the two sugars in the medium were consumed simultaneously, although glucose was consumed at a higher rate (Fig. 1). l-Rhamnose was only partially consumed, as observed in the l-rhamnose-only cultures, remaining approximately 31% of the initial content in the medium.

The major fermentation products on the d-glucose–l-rhamnose mixture corresponded to those observed for the d-glucose- and l-rhamnose-only fermentations, IBE and 1,2-propanediol, respectively. The concentration of 1,2-propanediol reached 78 mM, approximately four times higher than that seen in the l-rhamnose-only cultures, as a result of a higher sugar consumption.

As mentioned above, in the l-rhamnose-only cultures, low concentrations of propionic acid and *n*-propanol were detected in the medium (Table 1; Fig. 1). Remarkably, in cultures grown on mixtures of d-glucose and l-rhamnose, no propanol or propionate was detected (Table 1).

### l-Rhamnose pathway reconstruction.

**FIG 2:**
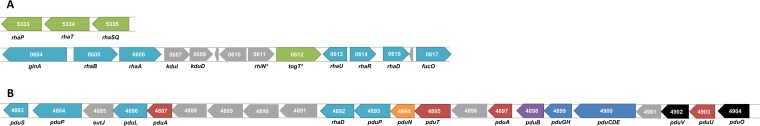
Schemes of the rhamnose utilization (A) and bacterial microcompartment (BMC) (B) clusters *in* C. beijerinckii DSM 6423. (A) The genes predicted to encode enzymes involved in the L-rhamnose metabolism are shown in blue, the genes in green encode putative l-rhamnose transporters, and the genes in gray are not reported to be involved in l-rhamnose metabolism. The functional homologue in Rhizobium leguminosarum bv. Trifolii is indicated for each gene. *, Homologues involved in rhamno-galacturonan catabolism were identified in Dickeya dadantii 3937. (B) BMC superlocus, compared to GRM3 (21). The functional equivalents in *Salmonella* Typhimurium of the genes predicted to be involved in the BMC are indicated below each gene. The genes are shown in different colors according to the function of the protein encoded: in blue, enzymes involved in the conversion of propanediol into propionate and propanol; in red, genes predicted to encode BMC-H shell proteins; in purple, genes for BMC-T shell proteins; in orange, genes for BMC-P shell proteins; and in gray, genes with unknown functions.

To further investigate the pathways of l-rhamnose metabolism in C. beijerinckii, bioinformatic analysis was performed on the genome sequence of the strain, as recently published (16, 18). Since the l-rhamnose catabolism was recently described for C. phytofermentans ISDg, this strain was used as main source of genes for query for BLASTp searches (19), but data on other organisms were used as well (15), as shown in Table 2. Genes encoding enzymes involved in all steps of the transport and metabolism of l-rhamnose into 1,2-propanediol could be identified (Table 2), with similarities ranging from 51% to 83%. As in C. phytofermentans, most genes involved in l-rhamnose catabolism were clustered within a genomic region (Fig. 2). For two of the enzymes encoded in the cluster, the rhamnulose phosphate aldolase enzyme (CIBE_0615) and the 1,2-propanediol oxidoreductase (CIBE_0617), gene duplications with high similarity were present (CIBE_3969 and CIBE_2890, respectively) elsewhere in the genome.

**TABLE 2:** Relative expression values from RNA-seq of C. beijerinckii DSM 6423 proteins putatively involved in l-rhamnose uptake and conversion

C. beijerinckii protein	Proposed protein function	Closest homologue with experimental evidence^*a*^		Log_2_ fold change in expression relative to d-glucose cultures		
		Organism	Protein (% similarity)	3 h	6.5 h	10 h
l**-**Rhamnose transport into the cell						
CIBE_5333	Rhamnose ABC transporter, permease subunit	Rhizobium leguminosarum bv. Trifolii	RhaP (51)	ND	4.18	3.96
CIBE_5334	Rhamnose ABC transporter, ATPase subunit	R. leguminosarum bv. Trifolii	RhaT (62)	1.26	4.47	4.11
CIBE_5335	Rhamnose ABC transporter, periplasmic solute binding subunit			3.14	5.9	4.47
CIBE_0612	MFS rhamnose cation symporter	Dickeya dadantii (Erwinia chrysanthemi)	TogT (63)	8.83	8.38	6.91
l-Rhamnose conversion to 1,2-propanediol						
CIBE_0605	Rhamnulokinase	C. phytofermentans	Cphy_1146 (73)	7.78	7.79	7.09
CIBE_0606	l-Rhamnose isomerase	C. phytofermentans	Cphy_1147 (78)	8.46	8.58	7.36
CIBE_0613	l-Rhamnose mutarotase	C. phytofermentans	Cphy_1149 (83)	ND	1.44	1.34
CIBE_0614	Transcriptional regulator	C. phytofermentans	Cphy_1187 (66)	1.8	1.46	1.55
CIBE_0615	Rhamnulose-1-phosphate aldolase	Escherichia coli (strain K-12)	RhaD (69)	6.99	7.37	6.84
CIBE_0617	l-1,2-Propanediol oxidoreductase	Escherichia coli (strain K-12)	FucO (79)	8.35	7.69	6.67

a All percent similarity values were determined using global alignments of protein sequences using the gapped BLAST algorithm (19). References for the characterized functional equivalent are as follows: Rhizobium leguminosarum bv. Trifolii rhamnose transporters, 15; C. phytofermentans rhamnose dissimilation enzymes, 12; Escherichia coli (strain K-12) rhamnulose-1-phosphate aldolase and l-1,2-propanediol oxidoreductase, 33 and 34; rhamnose MFS transporter, 15, 35, and 36.

Proteins putatively involved in l-rhamnose transport into the cell did not show similarity to those of C. phytofermentans but were most similar to those of an ABC transporter found in the soil bacterium Rhizobium leguminosarum and a transporter of the major facilitator superfamily (MFS) of the plant pathogen Dickeya dadantii (20).

A cluster of 21 genes contains the genes for the further conversion of 1,2-propanediol to propionic acid and *n*-propanol (Fig. 2). This cluster is almost identical to the clusters found in other organisms, including C. phytofermentans (13). 1,2-Propanediol is expected to be converted to propionaldehyde by a propanediol dehydratase. Unlike for *S*. Typhimurium, but similar to the case with C. phytofermentans, this is likely catalyzed by a vitamin B_12_-independent type of dehydratase (CIBE_4900; pduCDE). Propionaldehyde is further converted to propanol or propionyl coenzyme A (propionyl-CoA), catalyzed by a propanol dehydrogenase and a propionaldehyde dehydrogenase, respectively. A homologue for the propanol dehydrogenase is present (CIBE_4892), belonging to the Zn-dependent dehydrogenases. For the propionaldehyde dehydrogenase, 2 homologues are present in the cluster (CIBE_4884 and 4893), which is unlike the case for C. phytofermentans, which contains only one. Propionyl-CoA is converted to propionate involving a phosphate propionyl transferase and a propionate kinase. The cluster contains a gene encoding the transferase (CIBE_4886) but not one for the kinase. In C. phytofermentans, the propionate dephosphorylation is catalyzed by a kinase that is not specific to proniate-P, an acetate kinase which is encoded by a gene outside the l-rhamnose cluster. In C. beijerinckii, the bioinformatics analysis does not provide enough proof to identify the gene encoding the propionate kinase.

The BMC cluster identified in our C. beijerinckii strain belongs to the glycyl radical enzyme-containing microcompartment type (GRM) like the one described for C. phytofermentans (13). GRMs are found mainly in *Firmicutes* and some *Deltaproteobacteria* and *Olsenella* (21). This BMC locus type contains the metabolosome core enzymes and a glycyl radical enzyme which is the pyruvate lyase (CIBE_4900) in C. beijerinckii.

Bioinformatic studies (21, 22) showed that the GRM can be divided into subgroups dependent on the BMC shell proteins and the accessory genes that belong to the locus. The BMC cluster from C. beijerinckii belongs to the GRM.3 group because it contains genes that encode a peptidase, a flavoprotein, a EutJ homolog, *S*-adenosylmethionine synthetase, and signaling proteins. They are suspected to be involved in several metabolic pathways, such as vitamin B_12_ or *S*-adenosylmethionine synthesis.

### Transcriptome analysis.

Samples for mRNA isolation were taken from the fermentations on d-glucose and on l-rhamnose, as shown in Fig. 1. Time points for sampling were chosen in such a way that the early exponential, acidogenic, and solventogenic growth phases were represented. For d-glucose-grown cultures, samples were taken after 3.0, 5.0, and 9.5 h. For l-rhamnose-grown cultures, samples were taken after 3.0, 6.5, and 10 h. After RNA isolation and sequencing, the data were mapped against the recently sequenced genome of this C. beijerinckii strain to quantify gene expression levels under each condition (16, 18, 23).

In summary, mapping of the RNA-seq reads against the published genome of C. beijerinckii resulted in reliable reads in a range of 93.17% to 98.60% (Table S3) For analyzing differentially expressed genes, the TAMARA tool on the MicroScope platform was used (24). Thresholds were selected at |log_2_ (fold change)| > 3 and adjusted *P* value of <0.005, which resulted in a list of 671 significantly differentially expressed genes on l-rhamnose (11% of the genome). To see the impact of the l-rhamnose metabolism on selected functional clusters (Tables 2 and 3; Tables S4, S5, and S6) at all three time points, the log_2_ (fold change) was decreased to 0.5. Only fifty-nine of these significantly differentially expressed genes had a log_2_ fold change above 3.0 at the three time points and 115 genes at two time points. Out of these 59 genes, 25 correspond to the genes involved in l-rhamnose uptake and conversion and BMC formation. Moreover, except for the rhamnose mutarotase, the corresponding genes of the putative l-rhamnose-degrading enzymes were among the highest expressed during growth on l-rhamnose compared to glucose (Table 2). They were upregulated between 6.84 and 8.58 times on a log_2_-fold scale, depending on the enzyme and the sampling time point. For l-rhamnose transport two different transport systems were identified, an ABC and an MFS transporter. The transcript data show that both systems were indeed upregulated during growth on l-rhamnose. Especially, the transporter of the MFS type was highly upregulated (8.8-fold). As mentioned, for the rhamnulose phosphate aldolase two putative genes (*CIBE_0615* and *CIBE_3969*) were present. Of these only *CIBE_0615* was highly expressed on l-rhamnose, suggesting that *CIBE_0615* encodes the rhamnulose phosphate aldolase, which cleaves rhamnulose phosphate to lactaldehyde and dihydroxyacetone phosphate (DHAP). Likewise, two putative genes were proposed to encode the 1,2-propanediol oxidoreductase, which converts lactaldehyde to 1,2-propanediol. However, only *CIBE_0617* was highly expressed on l-rhamnose, indicating that this gene encodes the functional protein. Also, most putative genes for the conversion of 1,2-propanediol to propanol and propionate were highly expressed, confirming their role in l-rhamnose fermentation. For the propionaldehyde dehydrogenase, two genes were identified in the BMC cluster, and both were highly upregulated. As for the propionate kinase, since no gene encoding this enzyme is present in the BMC cluster, we looked at the differential expression of genes encoding kinases outside the BMC cluster. We noticed the upregulated expression a putative butyrate kinase (2-fold on a log_2_ scale), encoded by a gene present elsewhere in the genome (*CIBE_5515*). We can assume that this putative butyrate kinase acted as a propionate kinase when C. beijerinckii was grown on l-rhamnose.

**TABLE 3:** Composition and differential expression of the BMC locus in C. beijerinckii DSM 6423

C. beijerinckii protein	Proposed protein function	Closest homologue with experimental evidence^*a*^		Log_2_ fold change in expression relative to d-glucose cultures		
		Organism	Protein (% similarity)	3 h	6.5 h	10 h
CIBE_4883	Propanediol oxidoreductase	*S*. Typhimurium	PduS (68)	5.79	5.42	3.83
CIBE_4884	Propionaldehyde dehydrogenase	C. phytofermentans	Cphy_1178 (70)	9.49	8.32	6.24
CIBE_4885	EutJ, putative chaperonin, ethanolamine utilization protein	*S*. Typhimurium	EutJ (63)	8.62	7.61	6.57
CIBE_4886	Phosphate propanoyl transferase	C. phytofermentans	Cphy_1183 (67)	9.11	7.87	6.06
CIBE_4887	BMC-H shell protein	C. phytofermentans	Cphy_1182 (88)	8.73	7.91	5.63
CIBE_4888	Conserved membrane protein of unknown function			8.75	7.81	5.87
CIBE_4889	MetK, *S*-adenosylmethionine synthetase			9.12	8.08	6.58
CIBE_4890	Response regulator receiver protein			9.16	8.34	7.15
CIBE_4891	Signal transduction histidine kinase, LytS			9.42	8.49	6.93
CIBE_4892	Propanol dehydrogenase	Klebsiella pneumoniae	Dhat (43)	9.99	10	7.57
CIBE_4893	Propionaldehyde dehydrogenase	C. phytofermentans	Cphy_1178 (63)	9.54	9.56	7.27
CIBE_4894	BMC-P shell protein	C. phytofermentans	Cphy_1184 (71)	9.47	9.43	7.45
CIBE_4895	BMC-H shell protein	C. phytofermentans	Cphy_1186 (61)	10	10	7.8
CIBE_4896	Conserved protein of unknown function			11	10	8.15
CIBE_4897	BMC-H shell protein	C. phytofermentans	Cphy_1182 (89)	11	10	7.54
CIBE_4898	BMC-T shell protein	*S*. Typhimurium	PduB (67)	10	8.99	7.04
CIBE_4899	Propanediol dehydratase activator	Clostridium butyricum	DhaB2 (56)	11	11	7.69
CIBE_4900	Propanediol dehydratase	C. butyricum	DhaB1 (58)	12	10	6.94
CIBE_4901	Glutamine amidotransferase			6.3	5.94	5.44
CIBE_4902	Propanediol utilization protein	*S*. Typhimurium	PduV (62)	6.56	6.31	5.62
CIBE_4903	BMC-H shell protein	C. phytofermentans	Cphy_1176 (76)	7.67	6.72	5.73
CIBE_4904	Propanediol utilization protein	*S*. Typhimurium	PduO (62)	7.11	6.14	5.01
CIBE _4905	Hypothetical protein			2.15	ND	ND
CIBE _4906	Xanthine/uracil/vitamin C permease			−2.26	−3.31	1.65

a All percent similarity values were determined using global alignments of protein sequences using the gapped BLAST algorithm (19). References for the characterized functional equivalents are as follows: *Salmonella* Typhimurium, 11; C. phytofermentans, 12; Klebsiella pneumoniae, 37; *C. butyricum*, 38; for the BMC shell protein types, 39.

All the genes from the BMC locus were among the highest expressed when C. beijerinckii was grown on l-rhamnose (Table 3). Very low expression of this cluster was detected in glucose-grown cultures, from 4 to 55 reads per genes, compared to 234 to 81,830 reads in l-rhamnose-grown cultures.

**FIG 3:**
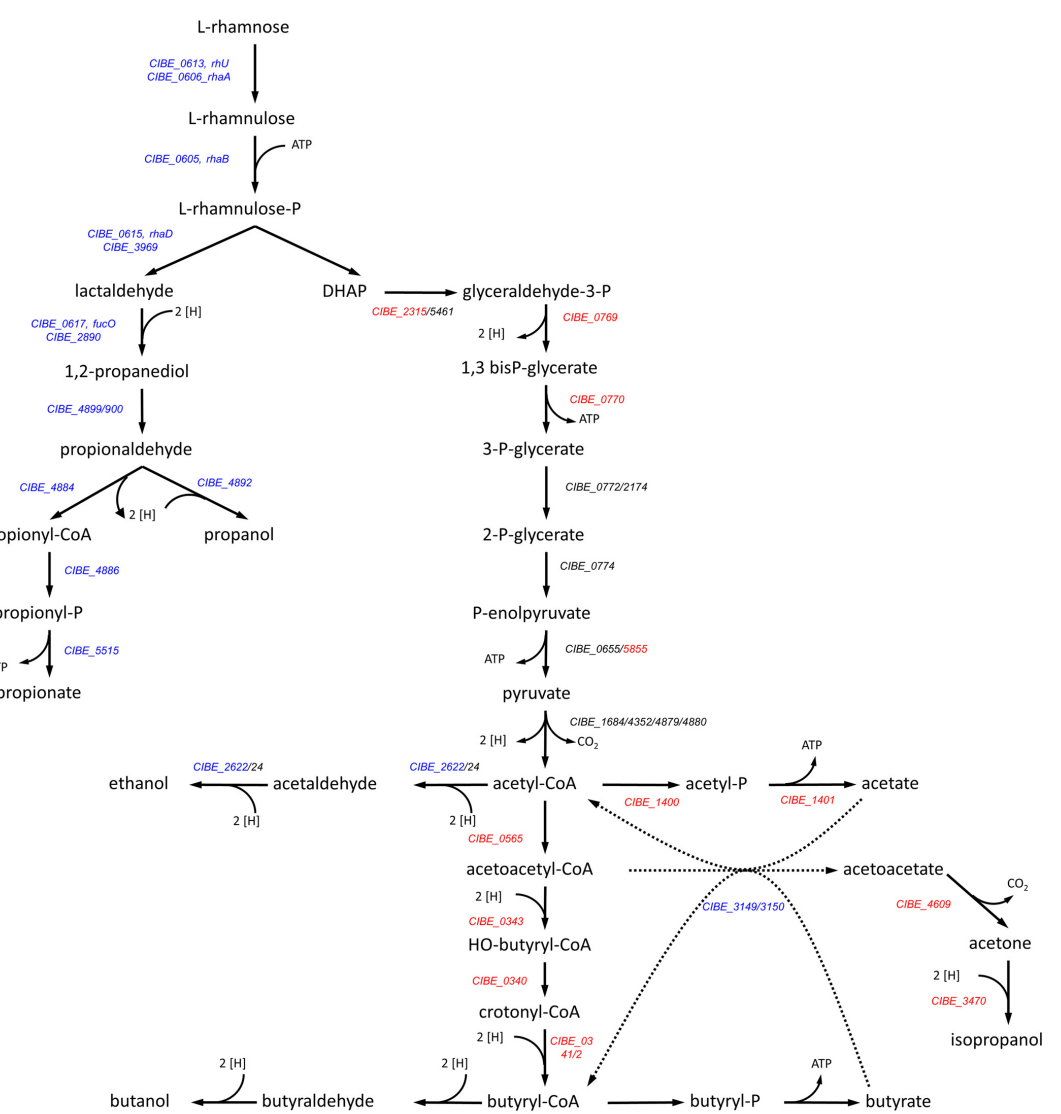
Model for the metabolic pathway of l-rhamnose by C. beijerinckii. The gene functions are based on sequence homology as shown in Tables 2 and 3 for the l-rhamnose-metabolizing pathway and on data from Máté de Gérando et al. (16) for the other routes. Genes shown in blue are overexpressed on l-rhamnose compared to glucose. Genes in red showed lower expression in l-rhamnose cultures than in glucose cultures.

As the l-rhamnose cultures produced acetate and butyrate, but no IBE, the expression of the main genes associated with glycolysis, acidogenesis, and solvent production were also analyzed. Most of the genes of the central metabolism to solvents were found to be less expressed in the l-rhamnose cultures (Fig. 3; Table S5). Genes predicted by Máté de Gérando et al. to code for enzymes involved in acidogenesis (16) were also less expressed in l-rhamnose cultures, suggesting that the reactions for acetate or butyrate production from l-rhamnose might be catalyzed by different enzymes. Genes involved in solvent formation (butanol and ethanol), however, were slightly upregulated during the early exponential growth phase of the l-rhamnose cultures. In Table S5, a list of differentially expressed genes encoding enzymes or other proteins predicted to be involved in glycolysis, acidogenesis, and solventogenesis is shown, including the fold change in expression between l-rhamnose and d-glucose.

The gene predicted to encode Spo0A, the global regulator of the metabolism in solventogenic clostridia (CIBE_2041), did not show significant difference in expression levels under growth on d-glucose or on l-rhamnose. This indicates that stationary-phase processes, including sporulation and stress response mechanisms, might not be differently regulated under both conditions. In Table S6, a list of predicted proteins related to sporulation and to stress response and their fold change in expression during growth on l-rhamnose compared to d-glucose are shown. Most genes encoding sporulation-related proteins or enzymes did not show important expression changes. However, data on the gene expression at stationary phase, after 20 h of fermentation, are needed to gain a better insight on differences in the regulation of stress response between d-glucose- and l-rhamnose-grown cultures.

## DISCUSSION

Next to lignocellulosic biomasses, aquatic biomasses such as seaweeds are a promising source for various industries (5, 17). It has been shown that clostridial species are able to grow on hydrolysates from the green seaweed U. lactuca and that the main products are acetone, butanol, and ethanol (ABE), which can be used in the biofuel industry (7). In addition, it was observed that 1,2-propanediol was produced as a result of l-rhamnose utilization. The metabolism of l-rhamnose was studied in more detail in various organisms, including E. coli and *Salmonella* Typhimurium (11, 25). In addition, Forsberg et al. showed already in the 1980s that several clostridial species were able to ferment l-rhamnose, and a fermentation pathway homologous to the one in E. coli and *Salmonella* Typhimurium was suggested (9). Recently, the production of propionate and propanol from l-rhamnose was demonstrated in C. phytofermentans and a fermentation model was proposed, which also included a specific organelle, the BMC (12). In the frame of this work, the l-rhamnose metabolism in the solventogenic strain C. beijerinckii DSM 6423 was investigated by genome analysis, fermentation studies, and transcriptomics.

C. beijerinckii DSM 6423 was tested for growth on U. lactuca hydrolysate, in an approach similar to that earlier described by our laboratory for the strain C. beijerinckii NCIMB 8052 (17). In contrast to the latter strain, C. beijerinckii DSM 6423 did not grow well on the pure hydrolysate. When the hydrolysate was diluted, the growth and sugar consumption improved, indicating that this strain could be inhibited by components of the hydrolysate. The content of elements that take part in salts, as potential inhibitors of growth, in the hydrolysate was estimated based on the data from Bikker and coworkers (17). The calculated content of elements the U. lactuca hydrolysate is shown in Table S2; S was the most abundant, with a concentration of 304 mM. The effect of salts on the growth of solventogenic clostridia is not well characterized yet, and only a few reports can be found on this topic. Ezeji et al. showed that levels of S corresponding to 93 mM in the form of sodium sulfate resulted in inhibitory effects on growth of C. beijerinckii on control media (26). The supplementation of microalga-derived hydrolysates with sodium chloride (NaCl) at 342 mM and higher resulted in inhibition of growth of Clostridium pasteurianum (27). In a different study, it was shown that the removal of S, among other elements, from a wood hydrolysate increased fermentability by C. beijerinckii (28). The strain used in this study showed higher sensitivity to inhibitors in the hydrolysate than strain NCIMB 8052, although the two are genetically very similar. Interestingly, the hydrolysate was rich enough in nutrients, and supplementation with nutrients was not required for growth.

On control medium, C. beijerinckii DSM 6423 was capable of growth on l-rhamnose as the sole source of carbon and energy. l-Rhamnose was converted into acetate, butyrate, and the typical l-rhamnose-derived products 1,2-propanediol, propanol, and propionate. Remarkably, typical solvents like isopropanol, butanol, and ethanol were not produced. Possibly, solvent production may not be necessary during l-rhamnose fermentation, as all reducing equivalents are required for the production of 1,2-propanediol and propanol (Fig. S2). Indeed DHAP (46.4 mM) conversion to acetyl-CoA leads to the formation of 46.4 mM NADH and 46.4 mM reduced ferredoxin. Reduced ferredoxin cannot directly donate electrons for solvent production but should first transfer its electrons to NAD. Assuming that all ferredoxin is converted to NADH, this would yield 92.8 mM NADH in total. This NADH is then used for the production of 46.4 mM 1,2 propanediol and 10.4 mM propanol (7.8 mM NADH is derived from propionaldehyde conversion) in the lactaldehyde branch and the production of 15.5 mM hydroxybutyryl-CoA and 15.5 mM butyryl-CoA in the DHAP branch (Fig. 3; Fig. S2). Thus, this leaves no reducing equivalents for solvent formation, which agrees with the absence of IBE production during l-rhamnose fermentation. Inside the BMC, propionaldehyde is either oxidized or reduced to propanol and propionyl-CoA. If both products were produced in equimolar amounts, no net NADH would be produced/consumed. However, the nonequal production of propanol (18.2 mM) and propionate (7.8 mM) indicates that some NADH must be come from outside the BMC, suggesting that reductant (NADH) is able to pass the BMC shell. It has been proposed before that NAD(H) is able to cross the BMC via specific pores (29).

From the metabolic pathway shown in Fig. 3, based on the work of Máté de Gérando et al. (16), it can be estimated that from 1 mol of l-rhamnose; 1 mol of 1,2-propanediol and 1 mol of DHAP should be formed. The 1,2-propanediol is then further metabolized into *n*-propanol and propionic acid. Table 1 shows that from 46.4 mM l-rhamnose consumed, 43.2 mM total products derived from 1,2-propanediol are formed, *viz*., 17.2 mM 1,2 propanediol, 18.2 mM *n*-propanol, and 7.8 mM propionic acid. DHAP is converted along the Embden-Meyerhof-Parnas (EMP) pathway to pyruvate, which is further metabolized into acetic acid (19.2 mM) and butyric acid (11.7 mM). These numbers are in agreement with the expected stoichiometry of this part of the pathway, as 19.2 mM acetate and 11.7 mM butyrate are derived from 42.6 mM DHAP (19.2 mM + 2 × 11.7 mM). However, some carbon that is not included in these calculations should end up in biomass. Thus, from 46.4 mM l-rhamnose (−2.1 mM biomass), 44.3 mM lactaldehyde and DHAP are produced. Based on the fermentation data, the following equations can be composed for both branches:

1 lactaldehyde→0.39 1,2 -propanediol+0.41 propanol+0.18 propionate

1 DHAP→0.43 acetate+ 0.26 butyrate (derived from 0.52 acetyl-CoA)

In accordance, the carbon and electron recovery both reached 96% for the l-rhamnose cultures. For the d-glucose and the d-glucose–l-rhamnose mixture, the recoveries were also high, at 95% and 88%, respectively.

Growth on l-rhamnose, however, stops before all l-rhamnose is converted. The reason for this is not clear. Possibly, too many acids are produced, which may become toxic. Commonly, during growth on d-glucose, solvents are produced to prevent excessive production of (undissociated) weak acids. However, running the fermentation under pH-controlled conditions did not improve the l-rhamnose conversion. During cofermentation of l-rhamnose and d-glucose, substantially more l-rhamnose is fermented, suggesting that there might be an energetic reason for the growth retardation on pure l-rhamnose. Theoretically, solventogenic d-glucose fermentation yields ∼2 mol of ATP per mol of sugar (30), which is more than twice the amount that can be obtained on l-rhamnose (0.9 mol of ATP/mole sugar), assuming that l-rhamnose uptake requires 1 ATP/mol of sugar (Fig. S2). The OD_600_ data show that growth is best on d-glucose (OD_600_ = 11.1), followed by the sugar mixture (OD_600_= 6.8) and the l-rhamnose culture (OD_600_ = 2.8). The lower growth yield on l-rhamnose correlates with the calculated ATP yield, which was approximately 38% of the yield on d-glucose (Table 1; Fig. S2). Moreover, l-rhamnose specifically induces the formation of the BMC (see below), whose protein shell may impose an extra biosynthetic energetic burden for the cell. The lower biomass yield on l-rhamnose than on d-glucose was observed earlier by Forsberg et al. (9), but its origin was not further studied then. However, despite this apparent difference in growth yields, the reason for the premature growth stop on l-rhamnose remains obscure.

The genome analysis revealed the presence of all necessary genes that are specifically needed for the anticipated enzymes of the l-rhamnose pathway. These include genes for l-rhamnose uptake and subsequent conversion to 1,2-propanediol, propanol, and propionate and of which many are clustered and probably organized in several operons. Also, various genes coding for shell proteins of the BMC were identified (Table 3). Sequence analysis of the different operons showed that the organization of the genes involved in l-rhamnose metabolism is similar to what was found in C. phytofermentans. Indeed, the genes responsible for l-rhamnose uptake and conversion are located in a different region than the BMC cluster. However, we observed significant differences in the genes involved in rhamnose transport and the size of the BMC cluster. In our strain, a gene coding for an l-rhamnose-specific ABC-type transporter was present in the genome, but another l-rhamnose-specific transporter gene belonging to the l-rhamnose conversion cluster (*CIBE_0612* [Table 2]) was highly upregulated during growth on l-rhamnose. Thus, the latter transporter is most likely responsible for l-rhamnose uptake. This transporter belongs to the major facilitator superfamily (MFS) type, which uses an H^+^ gradient to transport the sugar, described for Rhizobium leguminosarum bv. Trifolii. In solventogenic clostridia, this type of transporter has not been studied in detail yet.

The BMC cluster identified in C. beijerinckii DSM 6423 shows some differences from the one described for C. phytofermentans ISDg. It harbors 21 genes organized in nine operons in one locus, whereas a recent study shows that the C. phytofermentans genome harbors three BMC clusters, but only one was experimentally studied (12). The BMC gene cluster found in C. beijerinckii DSM 6423 is more related to the cluster found in other clostridia such as C. saccharolyticum K10 or C. ljungdahlii DSM 13528 and alphaproteobacteria, such as Rhodobacter capsulatus SB 1003. Homologues of the l-rhamnose utilization clusters found in C. beijerinckii DSM 6423 were also found in the genome of C. beijerinckii strain NCIMB 8052, which utilizes l-rhamnose as well (17).

Transcriptome analysis confirmed the involvement of the predicted genes in l-rhamnose conversion. Most metabolic proteins were highly upregulated (up to 8-fold). The only exception is propionate kinase. There was no specific propionate kinase gene identified by bioinformatic analysis. However, the upregulation (2-fold) of one of the copies of the butyrate kinase gene, *CIBE_5515*, in l-rhamnose-grown cells suggests that this gene may have activity toward propionyl-P. The various BMC shell proteins were also highly upregulated (9- to 12-fold compared to d-glucose-grown cells). Thus, the BMC was specifically induced during growth on L-rhamnose, as has also been described for l-rhamnose conversion in C. phytofermentans (13). On the l-rhamnose–d-glucose mixture we observed production of 1,2-propanediol but not of propanol and propionate. This suggests that d-glucose prevents induction of the BMC even when l-rhamnose is present.

It is assumed that the polyhedral shell prevents leakage of volatile metabolites or that it protects the cell against toxic intermediates, in this case propionaldehyde (31) or radicals of the 1,2-propanediol dehydratase reaction (13). As mentioned above, synthesis of the protein shell may exert a heavy burden on the protein synthesis machinery and may, therefore, also affect the growth rate and energetics of the cell.

In this study, we showed that C. beijerinckii is able to ferment l-rhamnose as a sole carbon and energy source, to produce acetic and butyric acids, 1,2-propanediol, propionic acid, and *n*-propanol, which are products of commercial interest. The metabolism of l-rhamnose in this strain shows similarities to pathways described for other clostridia but also presents interesting novelties, such as the presence of an MFS transporter for l-rhamnose. Cofermentation of l-rhamnose with d-glucose leads to higher l-rhamnose utlization, which shows potential for the use of this strain for fermentation of U. lactuca hydrolysates, or other l-rhamnose-containing streams, provided that salt toxicity can be reduced. The results of this study serve as a basis for further developments toward efficient biomass utilization for production of chemicals.

## MATERIALS AND METHODS

### Bacterial strains and culture conditions.

C. beijerinckii DSM 6423 was stored at −20°C as a spore suspension in 20% glycerol. The spore suspension was heat shocked for 1 min at 95°C before inoculation. Fermentations were performed in CM2 medium containing (in grams per liter) the following: yeast extract, 1.00; KH_2_PO_4_, 1.00; K_2_HPO_4_, 0.61; MgSO_4_·7 H_2_O, 1.00; FeSO_4_·7 H_2_O, 0.0066; *para*-aminobenzoic acid, 0.10; and ammonium acetate, 2.90. Stock solutions of d-glucose and l-rhamnose were autoclaved separately and added after autoclaving of the medium to a final concentration of 40 g liter^−1^. All liquid media were made anaerobic by flushing with nitrogen gas. Fermentations in a 400-ml working volume were performed in Infors HT Multifors bioreactors at 37°C and a stirrer speed of 150 rpm. Bacterial growth was monitored by measuring the optical density at 600 nm (OD_600_).

### Product analysis.

Fermentation substrates and products were measured by high-performance liquid chromatography (HPLC). Glucose, rhamnose, acetate, butyrate, lactate, acetone, ethanol, butanol, propanol, and isopropanol were measured in a Waters HPLC system equipped with a refractive index detector (Waters; model 2414) and a Shodex KC-811 300- by 8-mm column at 80°C with 3 mM H_2_SO_4_ as the mobile phase and a flow rate of 1.00 ml min^−1^. As an internal standard, 3 mM valeric acid in 1 M H_2_SO_4_ was used. Propionate and 1,2-propanediol were measured in a Dionex UltiMate3000 HPLC system equipped with a refractive index detector (Waters; model 2414) and a Bio-Rad Aminex HPX 87 H 300- by 8-mm column at 30°C with 3.7 mM H_3_PO_4_ as the mobile phase and a flow rate of 0.60 ml min^−1^. As an internal standard, 2.5 mM phthalic acid in water was used.

### Carbon recovery.

For the calculation of the carbon recovery, the total number of moles of carbon present in the products and biomass was divided by the total number of C moles of the substrates. Since acetate was present at the start and end of the fermentation, it was considered the substrate and product. For simplicity, acetate was considered the substrate when its final concentration was lower than at the start of the fermentation, whereas it was considered the product when its final concentration was higher than at the start. For d-glucose-grown cultures, the cell dry weight (cdw, in grams per liter) was calculated from the optical density at 600 nm using the following formula: cdw = OD_600_ × 0.28 + 0.13.

For the cultures grown on l-rhamnose and on the d-glucose–l-rhamnose mixture, the cell dry weight was determined by filtration of 10 ml of culture on a 0.22-μm porous filter, drying the biomass on the filter in an oven at 50°C overnight, and weighing.

The carbon content of biomass was calculated using the standard elemental biomass formula (CH_1.8_O_0.5_N_0.2_) given in reference 32. CO_2_ production during fermentation was taken into account. It was assumed that for the production of 1 mol of acetate or ethanol, butyrate or butanol, and acetone or isopropanol, 1, 2, and 3 mol of CO_2_ are produced, respectively.

### Electron recovery.

The electron recovery was determined by calculating the degree of reduction per mole of all compounds produced divided by all compounds produced. For simplicity, acetate was again considered the substrate when its final concentration was lower than at the start of the fermentation, whereas it was considered the product when its final concentration was higher than at the start. Since the production of H_2_ could not be accurately quantified during the fermentation, it was calculated from the stoichiometry of the reactions. The degrees of reduction per mole of substrate or product are as follows: d-glucose, 24; l-rhamnose, 26; acetate, 8; butyrate, 20; lactate, 12; acetone, 16; isopropanol, 18; butanol, 24; ethanol, 12; 1,2-propanediol, 16; propanol, 18; propionate, 14; and H_2_, 2. The degree of reduction of biomass was calculated from the standard elemental biomass composition of CH_1.8_O_0.5_N_0.2_, which corresponds to a degree of reduction of biomass of 21 electrons per mole.

### RNA sequencing.

Total RNA was isolated from C. beijerinckii DSM 6423 for transcriptome studies. Samples were taken from duplicate 400-ml fermentations from cells in early exponential, acetogenic, and solventogenic phases. Cells were pelleted for 15 min at 3,000 × *g* and 4°C and stored at −80°C until further use. RNA was isolated using TRIzol reagent (Thermo Fisher Scientific) and a PureLink RNA minikit (Thermo Fisher Scientific) according to the manufacturer’s protocol. In short, the cell pellet was thawed on ice and resuspended in 5 ml of TRIzol reagent for cell lysis. Next, 1 ml of chloroform was added, and after centrifugation for 15 min at 13,000 × *g*, the upper aqueous phase was mixed with an equal volume of 70% ethanol. The solution was loaded on a spin cartridge, washed once, and treated with 30 U of DNase I. After two additional washing steps, the RNA was eluted in RNase-free water. Quality and quantity of the isolated RNA were checked by gel electrophoresis and NanoDrop, respectively. Afterwards, the samples were stored at −80°C before being sent for sequencing. Library construction and sequencing were performed by Novogene Co. Ltd. mRNA was depleted with the Ribo-Zero magnetic kit, and a 250- to 300-bp insert cDNA library was constructed. Pair-ended 150-bp fragments were sequenced using the Illumina HiSeq platform. After sequencing, data were uploaded and analyzed with the MicroScope platform (24). Reads were mapped against the recently sequenced C. beijerinckii DSM 6423 genome (16).

### Accession number(s).

The DSM 6423 full genome sequence is available on the European Nucleotide Archive (ENA) under accession number PRJEB11626 (https://www.ebi.ac.uk/ena/data/view/PRJEB11626).

The DSM 6423 RNA-seq data described by Máté de Gérando et al. (16) were deposited in the NCBI BioProject Database under accession number GSE100024 (https://www.ncbi.nlm.nih.gov/geo/query/acc.cgi?acc= GSE100024) (23).

The DSM 6423 RNAseq data described here have been deposited in the ArrayExpress database at EMBL-EBI (https://www.ebi.ac.uk/arrayexpress) under accession number E-MTAB-7487.

## Supplementary Material

Supplemental file 1
