# Supplementary material for: l-Rhamnose Metabolism in Clostridium beijerinckii Strain DSM 6423
Source: Appl Environ Microbiol. 2019 Feb 20;85(5):e02656-18. doi: 10.1128/AEM.02656-18 (PMC6384099; doi:10.1128/AEM.02656-18)
Supplement: Supplemental file 1 [file AEM.02656-18-s0001.pdf]

Supplementary material

Diallo et al

Table S1. Fermentation of *Ulva lactuca* hydrolysates by *C. beijerinckii*. As controls, cultures on D-glucose, L-rhamnose, and a mix of D-glucose, L-rhamnose and D-xylose (G/R/X) were carried out. Codes: H-*Ulva*, hydrolysate of *Ulva lactuca* prepared as previously described (1); H-*Ulva* + N, indicates H-Ulva supplemented with nutrients as in CM2 medium; DH-Ulva indicates H-Ulva diluted 1:1 with demiwater; DH-*Ulva*+ N indicates H-Ulva+N diluted 1:1 with demiwater. In brackets, the times at which the last sample of the cultures was taken.

	Glucose (144h)	Rhamnose (144 h)	G/R/X (t=167h)	H-*Ulva* (144h)	H-*Ulva* +N (t=144h)	DH-*Ulva* (t=167h)	DH-*Ulva*+ N (t=167h)
**Substrates at t=0 (mM)**							
D-Glucose	209.2		141.0	114.7	102.9	57.3	51.4
L-Rhamnose		238.7	156.6	85.8	74.5	42.9	37.3
D-xylose			36.6	28.2	24.9	14.1	12.5
Acetate	36.5	35.0	36.1	9.8	46.5	4.9	23.2
**Substrates consumed (mM)**							
D-Glucose	164.3		141.0	21.9	21.3	52.9	50.3
L-Rhamnose		42.9	43.3			8.2	5.0
Xylose			34.6			6.8	7.1
Acetate*	23.6		5.5				10.2
**Products at end (mM)**							
Acetate*		17.7		27.1	60.9	17.5	
Butyrate	6.9	13.8	17.3	6.2	6.8	27.7	47.6
Isopropanol	31.9	nd	23.0	7.5	7.0	6.2	10.5
Ethanol	2.6	nd	6.3	nd	nd	nd	nd
Butanol	84.1	nd	72.7	13.2	10.1	21.3	36.8
1,2-Propanediol	nd	15.0	22.6	nd	nd	nd	0.8
Propanol	nd	19.0	nd	nd	nd	nd	nd
Propionate	nd	7.0	9.6	nd	nd	nd	nd
**Yields**							
mM IBE/mM (D-Glucose+D-xylose) consumed	0.72		0.58	0.94	0.80	0.46	0.82

*Acetate was present in the media as component, and in case it was utilized it is considered as a substrate. When acetate is produced, it is considered as a product and the end concentration in the cultures is indicated. “nd” indicates not detected.

Table S2. Salt content in the *U. lactuca* hydrolysate, calculated from the analysis data shown in Bikker et al (1).

Element	Concentration (mM)
K	73
Mg	185
Na	67
Cl	32
S	304

Table S3. Mapping overview of the performed RNA sequencing.

Sample	Time point [h]	Total number of reads	Number of unmapped reads	Number of reliable reads [%]
Glc_1	3	5,354,497	33,708	96.17
Glc_1	5	4,331,116	20,562	96.94
Glc_1	9.5	4,721,967	19,719	96.71
Glc_2	3	4,466,679	54,964	94.88
Glc_2	5	4,700,384	23,416	95.86
Glc_2	9.5	5,175,736	23,026	95.71
Rha_1	3	4,505,619	17,259	98.60
Rha_1	6.5	4,718,680	43,935	97.52
Rha_1	10	4,284,034	105,542	95.84
Rha_2	3	4,525,859	28,847	98.20
Rha_2	6.5	4,987,878	53,998	97.19
Rha_2	10	4,320,185	191,739	93.71

Table S4: Relative expression value of genes belonging to the L-rhamnose conversion cluster but involved in other pathways

***C. beijerinckii* protein**	**Predicted function**	**Log2 fold change in expression relative to D-glucose**		
		**3 h**	**6.5 h**	**10 h**
**chondroitin sulfate degradation / rhamnogalacturonan degradation**				
CIBE_0607	4-deoxy-L-threo-5-hexosulose-uronate ketol-isomerase, 5-keto-4-deoxyuronate isomerase	7.74	7.65	6.56
CIBE_0608	2-keto-3-deoxygluconate oxidoreductase	6.92	7.04	6.21
CIBE_0610	Glycosyl hydrolase, family 88, rhiN	6.65	6.57	6.43
**unknown function**				
CIBE_0609	protein of unknown function	6.22	6.67	6.89
CIBE_0611	Xylose isomerase domain protein TIM barrel	4.03	4.11	4.04

Table S5. Differential expression during growth on L-rhamnose relative to D-glucose of the genes involved in the central metabolism. The clusters and gene assignments were based on the transcriptome study of Mate de Gerando et al (2)

***C. beijerinckii* protein**	**Predicted function**	**Log2 fold change in expression relative to D-glucose cultures**		
		**3 h**	**6.5 h**	**10 h**
**Glycolysis**				
CIBE_0769	glyceraldehyde-3-phosphate dehydrogenase	-1.19	-2.55	-1.53
CIBE_2315	fructose-1,6-bisphosphate aldolase	-1.08	-1.99	-1.22
CIBE_0770	phosphoglycerate kinase	n.d	-2.45	-1.59
CIBE_0414	glucose-6-phosphate isomerase	n.d	-1.35	-1.12
CIBE_0771	triose phosphate isomerase	n.d	-2.41	-1.29
CIBE_5856	6-phosphofructokinase	-2.76	-2.16	n.d
CIBE_0774	enolase	1.33	n.d	n.d
CIBE_0772	phosphoglycerate mutase	n.d	-1.09	n.d
CIBE_1193	6-phosphofructokinase 2	4.98	2.33	n.d
CIBE_0655	Pyruvate kinase	n.d	-0.91	n.d
CIBE_0755	Phosphofructokinase	-2.3	n.d	n.d
CIBE_3600	Fructose-bisphosphate aldolase class 1	2.37	3.68	n.d
CIBE_5451	fructose-1,6-bisphosphatase	n.d	n.d	-0.82
CIBE_2174	Phosphoglycerate mutase	n.d	n.d	-0.96
CIBE_5855	pyruvate kinase	-3.44	-2.56	n.d
CIBE_5461	putative fructose-bisphosphate aldolase	1.17	1.55	1.07
CIBE_5876	putative transketolase N-terminal section	-1.5	-1.99	-1.37
**Pentose phosphate pathway**				
CIBE_5875	putative transketolase C-terminal section	-1.32	-1.87	-1.05
CIBE_0561	ribose 5-phosphate epimerase	n.d	-1.11	-1.04
CIBE_1389	ribulose-5-phosphate 3-epimerase	n.d	-1.4	-0.89
CIBE_3260	Transaldolase	2.82	n.d	n.d
CIBE_5350	putative transaldolase	1.86	n.d	n.d
CIBE_0719	ribose 5-phosphate epimerase	-1.53	n.d	1.84
CIBE_2821	putative transaldolase	2.02	1.3	-0.77
**Acidogenesis**				
CIBE_2950	Histidine kinase	1.1	n.d	n.d
CIBE_5186	Pyruvate-flavodoxin oxidoreductase	n.d	-2.73	-3.12
CIBE_0565	degradative acetoacetyl-CoA thiolase	-2.74	-3.26	-4.02
CIBE_0339	putative Methylglutaconyl-CoA hydratase	-2.28	-2.82	-3.84

C. beijerinckii protein	Predicted function	Log2 fold change in expression relative to D-glucose cultures		
		3 h	6.5 h	10 h
CIBE_6076	Acyl-CoA dehydrogenase	2.32	n.d	n.d
CIBE_0343	3-hydroxybutyryl-CoA dehydrogenase	-2.58	-3.06	-3.87
CIBE_1400	Phosphate acetyltransferase	n.d	-2.43	-1.68
CIBE_0224	Butyrate kinase	-2.73	-3.42	-2.78
CIBE_1401	acetate kinase	n.d	-2.3	-1.35
CIBE_0223	phosphate butyryl coenzyme A transferase	-2.64	-3.26	-2.69
CIBE_1204	pyruvate formate lyase I	-1.36	-1.81	-1.69
CIBE_1205	pyruvate formate lyase activating enzyme 1	n.d	n.d	-1.58
CIBE_4880	Pyruvate flavodoxin/ferredoxin oxidoreductase domain protein	2.83	3.43	-0.78
CIBE_4879	Pyruvate ferredoxin/flavodoxin oxidoreductase, beta subunit	2.85	3.57	-0.94
CIBE_3432	acyl-CoA dehydrogenase	3.26	2.42	-2.26
CIBE_4352	Pyruvate flavodoxin/ferredoxin oxidoreductase domain protein	2.04	1	n.d
CIBE_4218	acyl-CoA dehydrogenase	1.52	3.31	n.d
CIBE_0327	Electron transfer flavoprotein subunit beta	-2	n.d	-4.01
CIBE_0328	Electron transfer flavoprotein subunit alpha	-1.36	n.d	-3.71
CIBE_1564	putative beta-hydroxyacid dehydrogenase	n.d	-1.03	n.d
CIBE_4841	Butyrate kinase	1.73	1.35	1
CIBE_4329	acetyl-CoA acetyltransferase	n.d	-1.76	-1.48
CIBE_5515	Butyrate kinase	2.69	2.41	2.37
CIBE_1684	Pyruvate-flavodoxin oxidoreductase	1.16	n.d	2.75
CIBE_5454	putative Methylglutaconyl-CoA hydratase	1.81	3.92	1.44
CIBE_0340	acyl-CoA dehydrogenase	-2.6	-3.24	-4.03
CIBE_2634	putative hydroxyacid dehydrogenase	2.12	n.d	n.d
CIBE_5833	3-oxoacyl-(Acyl-carrier-protein) synthase III	-1.49	n.d	n.d
CIBE_5834	Acetoacetyl-CoA reductase	-1.5	n.d	n.d
**A/IBE production**				
CIBE_0565	degradative acetoacetyl-CoA thiolase	-2.74	-3.26	-4.02
CIBE_6076	Acyl-CoA dehydrogenase	2.32	n.d	n.d
CIBE_4607	putative aminoacyloate CoA-transferase	-3.67	-1.97	-4.87
CIBE_4609	Acetoacetate decarboxylase	-3.55	-1.3	-4.2
CIBE_3470	NADP-dependent isopropanol dehydrogenase	n.d	n.d	-4.63
CIBE_2050	NADPH-dependent butanol dehydrogenase	3.92	n.d	-1.75
CIBE_3149	putative acyloate-acetoacetate CoA-transferase	2.76	4.1	n.d
CIBE_3150	acetoacetyl CoA-transferase (subunit A)	3.97	4.28	n.d

C. beijerinckii protein	Predicted function	Log2 fold change in expression relative to D-glucose cultures		
		3 h	6.5 h	10 h
CIBE_3970	Acetate CoA-transferase YdiF	n.d	-1.2	-2.52
CIBE_5462	Iron-containing alcohol dehydrogenase	2.67	3.13	n.d
CIBE_2622	NADPH-dependent butanol dehydrogenase	4.47	3.53	1.44
CIBE_2354	Alcohol dehydrogenase	-1.31	n.d	n.d
CIBE_5453	short chain acyl-CoA transferase: fused alpha subunit ; beta subunit	1.11	3.13	1.67

Table S6: Differential expression of the genes involved in other stationary phase processes. For stress response genes, a comparison was made with data from (3)

C. beijerinckii **protein**	**predicted function**	**Log2 fold change in expression in L-rhamnose relative to D-glucose**		
		**3 h**	**6.5 h**	**10 h**
**sporulation**				
CIBE_0044	putative 2-phosphosulfolactate phosphatase	-1.57	n.d	n.d
CIBE_0055	Spore maturation protein	n.d	2.27	-0.78
CIBE_0056	spore maturation protein	n.d	2.37	n.d
CIBE_0064	conserved protein of unknown function	1.77	3.72	n.d
CIBE_0089	regulator required for spore cortex synthesis (stage V sporulation)	1.58	n.d	-0.86
CIBE_0098	transcriptional regulator	2.38	1.22	2.6
CIBE_0099	Stage V sporulation protein B	n.d	2.99	n.d
CIBE_0103	YabP family protein	n.d	2.74	n.d
CIBE_0104	Spore cortex biosynthesis protein YabQ	n.d	3.09	n.d
CIBE_0107	Sporulation protein, spoIIE	n.d	4.12	-2.79
CIBE_0132	subunit of a sporulation, competence and biofilm formation regulatory complex	n.d	-1.02	-1.1
CIBE_0218	Cell wall hydrolase, SleB	n.d	4.03	n.d
CIBE_0504	Histidine kinase	1.69	n.d	n.d
CIBE_0535	Spore coat protein CotS	1.43	2.72	2.16
CIBE_0537	conserved protein of unknown function	2.66	5.23	n.d
CIBE_0549	Sporulation stage II, protein R	-1.81	n.d	n.d
CIBE_0578	Peptidase M23B	1.34	5.61	n.d
CIBE_0579	transcriptional regulator	n.d	5.57	n.d
CIBE_0641	Small, acid-soluble spore protein 1	-2.02	n.d	1.94
CIBE_0672	Peptidase M50	n.d	3.72	0.83
CIBE_0730	conserved protein of unknown function	1.37	n.d	n.d
CIBE_0739	Stage V sporulation protein R	3.38	4.62	n.d
CIBE_0761	Histidine kinase	n.d	n.d	-0.77
CIBE_0992	Anti-sigma F factor antagonist	n.d	n.d	-3.08
CIBE_0994	RNA polymerase sporulation-specific sigma factor (sigma-F)	n.d	n.d	-2.4
CIBE_1003	Stage II sporulation protein P	n.d	4.23	-1.01
CIBE_1023	putative stage IV sporulation YqfD	n.d	n.d	-0.88
CIBE_1036	Spore coat protein CotS	2.2	5.72	n.d
CIBE_1351	RNA polymerase sporulation-specific sigma-29 factor (sigma-K)	2.85	3.13	1.72
CIBE_1355	Peptidase U4, Sporulation sigma-E factor-processing peptidase	n.d	3.04	-2.12
CIBE_1356	RNA polymerase sporulation-specific sigma-29 factor (sigma-E)	n.d	3.5	-2.17

C. beijerinckii protein	predicted function	Log2 fold change in expression in L-rhamnose relative to D-glucose		
		3 h	6.5 h	10 h
CIBE_1357	RNA polymerase sporulation-specific sigma factor (sigma-G)	1.44	3.86	-1.9
CIBE_1358	essential sporulation protein	2.07	4.11	-1.47
CIBE_1362	Multi-sensor signal transduction histidine kinase	n.d	n.d	-0.75
CIBE_1373	morphogenetic stage IV sporulation protein	3.23	5.13	-1.14
CIBE_1376	essential sporulation DNA binding protein; regulator of biofilm formation	n.d	n.d	-0.97
CIBE_1398	Sporulation integral membrane protein YlbJ	n.d	3.96	n.d
CIBE_1448	Sporulation protein YlmC/YmxH	n.d	1.86	n.d
CIBE_1451	spore DNA translocase	1.21	n.d	-0.86
CIBE_1456	regulator required for dehydratation of the spore core and assembly of the coat (stage V sporulation)	5.93	2.02	1.96
CIBE_1525	Sporulation protein	2.39	5.12	3.69
CIBE_1586	conserved protein of unknown function	2.69	5.34	5.4
CIBE_1605	Sporulation protein SpoVB	n.d	4.03	n.d
CIBE_1674	Small, acid-soluble spore protein beta	4.74	1.55	4.77
CIBE_1733	putative spore coat protein	n.d	n.d	1.8
CIBE_1735	Spore coat protein	1.61	n.d	1.78
CIBE_1736	putative spore coat protein	n.d	n.d	1.67
CIBE_1789	Multi-sensor signal transduction histidine kinase	2.09	n.d	n.d
CIBE_1807	Sporulation protein	1.61	2.54	3.03
CIBE_1819	Stage V sporulation protein D	1.59	n.d	-1.39
CIBE_1821	Stage V sporulation protein D	n.d	n.d	-1.4
CIBE_1825	factor for spore cortex peptidoglycan synthesis (stage V sporulation)	n.d	n.d	-0.94
CIBE_1845	Acetyltransferase	-1.29	n.d	n.d
CIBE_2020	ATP-binding stage III sporulation protein	n.d	4.02	-1.77
CIBE_2021	Stage III sporulation protein AB	n.d	4.43	-1.66
CIBE_2022	Stage III sporulation AC family protein	n.d	4.76	-1.35
CIBE_2023	stage III sporulation protein	n.d	5.52	-1.25
CIBE_2024	Sporulation stage III, protein AE	1.15	6.35	-1.13
CIBE_2025	Sporulation stage III, protein AF	n.d	6.01	-1.37
CIBE_2026	Stage III sporulation protein AG	n.d	5.54	-1.94
CIBE_2027	Stage III sporulation protein AH	1.88	5.67	-1.55
CIBE_2041	Sporulation protein spo0A	1.24	n.d	-1.43
CIBE_2218	D-alanyl-D-alanine carboxypeptidase DacF	1.62	n.d	3.79
CIBE_2221	Uncharacterized spore protein YtfJ	1.44	2.69	1.53
CIBE_2227	Transcriptional regulator, MerR family	n.d	-1.2	-0.92
CIBE_2390	Sporulation stage II, protein M	3.55	7.3	0.99
CIBE_2490	component of the inner spore coat	3.64	6.62	5.17

C. beijerinckii protein	predicted function	Log2 fold change in expression in L-rhamnose relative to D-glucose		
		3 h	6.5 h	10 h
CIBE_2491	Spore coat peptide assembly protein CotJB	4.4	7.62	5.75
CIBE_2492	conserved protein of unknown function	5.56	8.1	5.65
CIBE_2495	Histidine kinase	n.d	n.d	-0.99
CIBE_2581	dihydroxyacetone kinase, N-terminal domain	1.77	2.61	1.14
CIBE_2775	Small, acid-soluble spore protein beta	4.31	1.43	1.65
CIBE_2875	PAS/PAC sensor signal transduction histidine kinase	1.99	n.d	1.16
CIBE_2913	Small, acid-soluble spore protein beta	3.53	3.43	3.92
CIBE_2950	Histidine kinase	1.1	n.d	n.d
CIBE_3040	conserved protein of unknown function	2.09	5.15	5.24
CIBE_3507	conserved protein of unknown function	3.59	5.3	1.53
CIBE_3532	Transcriptional regulator, AbrB family	1.96	1.79	-1.11
CIBE_3643	Histidine kinase	1.8	n.d	n.d
CIBE_3647	Sensor histidine kinase	1.19	n.d	n.d
CIBE_3649	Small acid-soluble spore protein, alpha/beta type	n.d	n.d	4.72
CIBE_3698	Small acid-soluble spore protein, alpha/beta type	4.67	2.07	5.58
CIBE_3923	Spore cortex-lytic protein	3.74	3.76	0.91
CIBE_3935	Small, acid-soluble spore protein beta	2.23	n.d	4.09
CIBE_3951	Spore protein	2.93	n.d	3.68
CIBE_3965	Spore protein	2.79	1.89	5.91
CIBE_4004	conserved protein of unknown function	1.37	n.d	n.d
CIBE_4085	Transcriptional regulator, AbrB family	2.9	n.d	1.61
CIBE_4156	conserved exported protein of unknown function	3.83	n.d	1.19
CIBE_4576	phosphatase	1.04	n.d	n.d
CIBE_4975	18 kDa heat shock protein	1.48	n.d	0.98
CIBE_5092	Sporulation protein YunB	n.d	2.48	n.d
CIBE_5119	component of the flagellar export machinery	1.41	n.d	n.d
CIBE_5516	Protein Tlp homolog	3.06	n.d	5.34
CIBE_5860	putative morphogen	n.d	-0.92	-1.02
CIBE_5891	Transcriptional regulator, AbrB family	-1.81	n.d	1.42
CIBE_6123	chromosome partitioning protein; transcriptional regulator	n.d	n.d	-0.75
CIBE_6124	DNA-binding protein Spo0J-like	n.d	n.d	-1.07
CIBE_b0010	protein of unknown function	5.24	2.27	2.44
CIBE_0993	anti-sigma factor (antagonist of sigma(F)) and serine kinase	n.d	n.d	-2.9
**stress response**				
CIBE_0139	class III stress response-related ATPase, AAA+ superfamily	0.94	n.d	n.d
CIBE_0289	Rubrerythrin	2.95	n.d	-0.85
CIBE_0347	chaperonin small subunit	2.36	n.d	n.d

C. beijerinckii protein	predicted function	Log2 fold change in expression in L-rhamnose relative to D-glucose		
		3 h	6.5 h	10 h
CIBE_0348	chaperonin large subunit	2.44	n.d	n.d
CIBE_0740	Rubrerythrin	3.11	4.87	n.d
CIBE_2142	33 kDa chaperonin	-1.22	-1.06	n.d
CIBE_1008	Heat-inducible transcription repressor HrcA	1.35	n.d	n.d
CIBE_1009	Protein GrpE	1.29	n.d	n.d
CIBE_1010	molecular chaperone	1.6	n.d	n.d
CIBE_1502	Lon protease	1.23	n.d	n.d
CIBE_1581	ATP-dependent Clp protease proteolytic subunit	n.d	n.d	-0.96
CIBE_1740	Superoxide dismutase [Fe]	1.71	n.d	n.d
CIBE_2199	Superoxide dismutase [Mn/Fe]	1.75	3.48	0.89
CIBE_2745	Reverse rubrerythrin-1	1.6	2.08	2.62
CIBE_2934	Reverse rubrerythrin-1	4.38	n.d	n.d
CIBE_3517	Rubrerythrin	4.54	5.08	0.77
CIBE_3635	Rubrerythrin	-1.39	n.d	n.d
CIBE_4975	18 kDa heat shock protein	1.48	n.d	0.98
CIBE_3943	Rubrerythrin	2.41	3.1	2.43

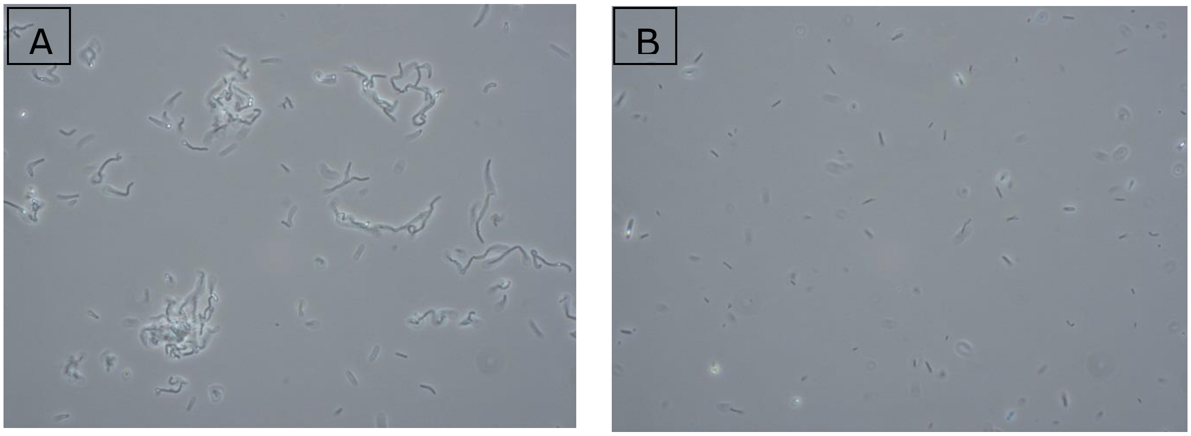

Figure S1. Phase-contrast photographs (1000x) of cultures grown on a glucose/rhamnose mix (A) and on rhamnose (B) after 30 h. of fermentation.

Diallo et al

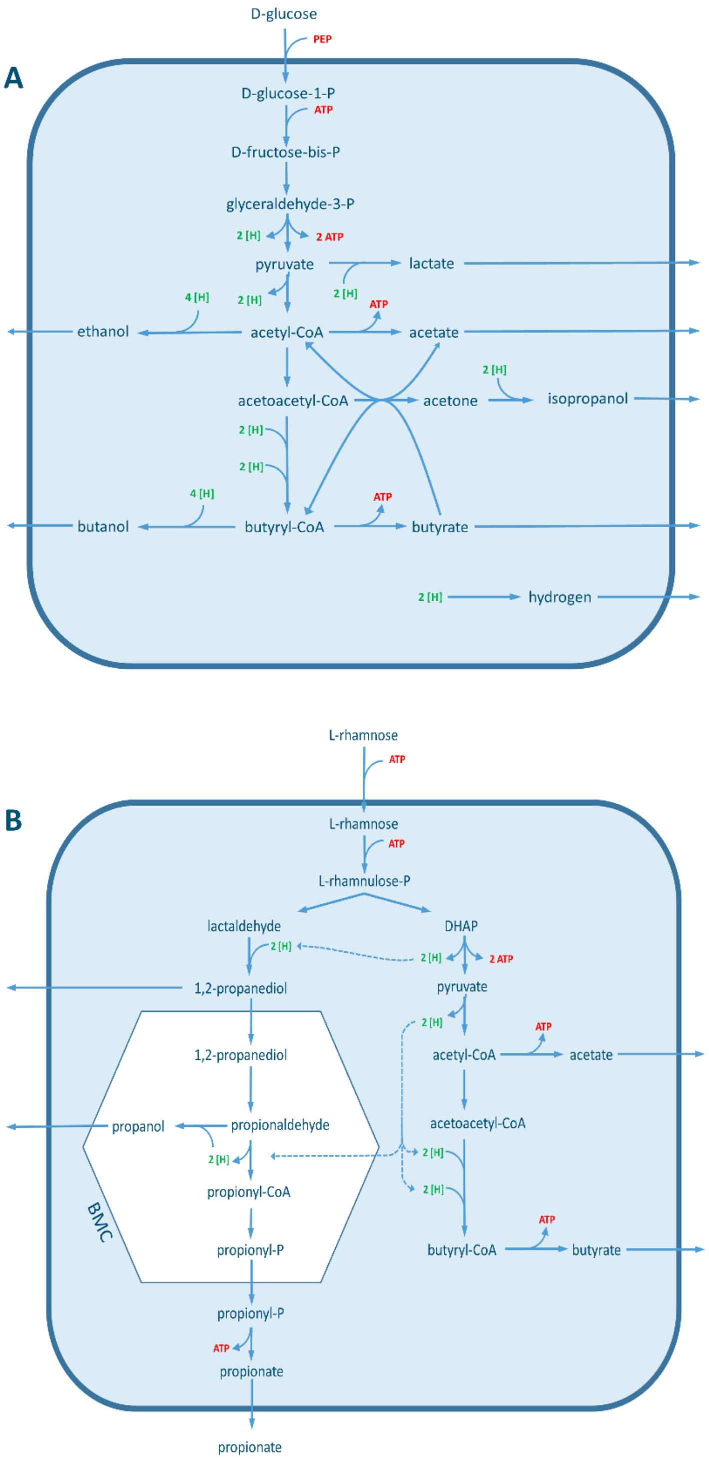

Figure S2. Redox balance and energy metabolism in C. beijerinckii. A, growth on D-glucose and **B**, growth on L-rhamnose.

References

1. Bikker P, Krimpen MMv, Houweling-Tan B, Huijgen WJJ, Scaccia N, Wikselaar Pv, Hal JWv, López-Contreras AM. 2016. Biorefinery of the green seaweed *Ulva lactuca* to produce chemicals, biofuels and animal feed. J Appl Phycol 28:3511-3525.
2. Máté de Gérando H, Wasels F, Bisson A, Clement B, Bidard F, Jourdier E, López-Contreras AM, Lopes Ferreira N. 2018. Genome and transcriptome of the natural isopropanol producer *Clostridium beijerinckii* DSM 6423. BMC genomics 19:242.
3. Saujet L, Monot M, Dupuy B, Soutourina O, Martin-Verstraete I. 2011. The key sigma factor of transition phase, SigH, controls sporulation, metabolism and virulence factor expression in *Clostridium difficile*. J Bacteriol doi: 10.1128/JB.00272-11 JB. 00272-11.
